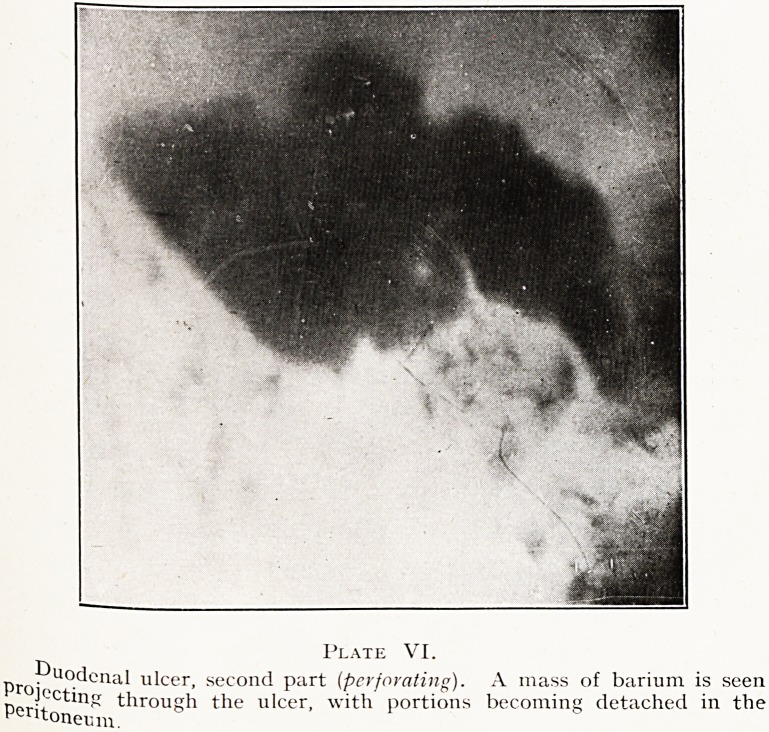# Duodenal Ulcers : Their Detection by Photography

**Published:** 1924-01

**Authors:** T. Carwardine

**Affiliations:** Surgeon to the Bristol Royal Infirmary


### DUODENAL ULCERS:
### THEIR DETECTION BY PHOTOGRAPHY.

BY

T. Carwardine, M.S.,

*Surgeon to the Bristol Royal Infirmary.*

---

In a recent paper on peptic ulcer in this *Journal* it was emphasised that X-ray diagnosis is destined to exceed in precision all other evidences.[1] We may now record our experiences in the detection of duodenal ulcers by photography of X-ray films, as well as some of the conclusions arrived at.

When an ulcer-bearing area of the duodenum is viewed sideways after an opaque meal there may be visible a wedge-shaped area in which the barium is absent, with a central dark spot or depression above representing the actual ulcer, undulations in its neighbourhood, a shaded margin, and an interval or notch below. These points will be seen in Plate I. It is a key to the interpretation of other photographic signs if the reader will imagine what the features would be if they were viewed from above instead of from the side.

When seen from above, like an architect's plan, the ulcer-bearing area will be found to give three signs, which, taken together, I regard as diagnostic of a duodenal ulcer. They are (1) a central spot, (2) a mottled halo, (3) a surrounding penumbra. All three signs are apparent in Plate II. A particularly distinct small area of spasm is visible near the left lower part which resembles a pendant in form; and the contiguous margin of the dark duodenal cap is irregular, due to small depressions and elevations.

PLATE I.

Duodenal ulcer, side view. Note the central depression above, the undulations, and shaded periphery.

PLATE II.

Duodenal ulcer, face view. Note the central spot, the mottled halo, and the penumbra.

PLATE III.

Duodenal ulcer, three-quarter face view. Note the central spot and penumbra.

PLATE IV.

Duodenal ulcer, seen sideways but vertically, giving appearance of duodenal deformity.

A modification of the foregoing may be observed when the ulcer is disposed somewhat sideways—a three-quarter face view, instead of a full-face one. In such event the penumbra appears half-moon shaped or crescentic, with a central spot. We have diagnosed duodenal ulcer from such a photograph correctly when the clinical evidences were rather in favour of gall-bladder trouble. (Plate III.)

The shape and position of the duodenal bulb or cap are not constant, although generally compared to Napoleon's cap, and situated on the right of the spine. Deformities of the duodenal contour, combined with retention and hyper-peristalsis in a large but otherwise normal stomach, are usually regarded as trustworthy indications of duodenal ulcer.[2] Our observations lead us to the conclusion that the deformity of the duodenal contour of a zig-zag form to the right of the spine is due to an ulcer-bearing area being seen sideways and vertically, not horizontally as in the first example described and illustrated. (Plate IV.)

A key to the interpretation of the appearances will be found in Figs. 1, 2, 3 and 4, illustrating an ulcer seen sideways, horizontally; the full-face view of an ulcer, such as one on the anterior wall of the duodenum; a three-quarter face view; and an ulcer seen sideways, vertically. when spasm or deformity of the duodenal contour is conspicuous.

In the interpretation of these signs one should be familiar with other deformities of the duodenum: concentric around duodenal bands—angularity and perhaps concavity from adhesion to the gall-bladder—irregular spasm, sometimes very distinct, in appendix dyspepsia—a generalised duodenitis from multiple ulcers—the shaggy duodenal outline in acute pancreatitis—and the like. Experience will enable one to differentiate such deformities from those associated with duodenal ulcer, as well as to detect duodenal ulcers by the more direct method of actual photography, especially when the characteristic central spot, mottled halo, and penumbra are present together.

Ulcers in the second part of the duodenum are rare, and they may give different appearances to those described, particularly if adherent to the gall-bladder. A film taken immediately after a barium meal showed a large ulcer in

FIG. 1.

Side view, horizontal.

FIG. 3.
Three-quarter face view.

FIG. 2.

Full-face view.

FIG. 4.

Side view, vertical.

*These figures represent diagrammatically the appearances seen in Plates* I., II., III., IV.

this situation. (Plate V.) A second photograph taken an hour later showed a mass of barium projecting through the ulcer, with small portions becoming detached, owing to subacute perforation—confirmed by operation. (Plate VI.)

The method adopted in these investigations was to take a reduced positive impression of the film on a lantern plate by reduction for general purposes, and for special purposes—

Plate V.
Duodenal ulcer, second part, adherent to gall-bladder, before perforating.

Plate VI.
Duodenal ulcer, second part (*perforating*). A mass of barium is seen projecting through the ulcer, with portions becoming detached in the peritoneum.

as in the half-tone plates accompanying this paper—to take a positive impression from the film by contact, with subsequent intensification.

I wish to acknowledge the help I have received from Dr. F. J. A. Mayes, Radiologist to the Bristol Royal Infirmary, who has prepared the films from which the photographs were taken.

REFERENCES.

[1] T. Carwardine, *Bristol M.-Chir. J.*, 1923, xl., 71.

[2] R. D. Carman, *Collected Papers of the Mayo Clinic :* "The Roentgen diagnosis and localization of peptic ulcer," xii., 1920 ; "Errors in the Roentgenologic diagnosis of duodenal ulcer," xiii., 1921.

---

## Figures and Tables

**Plate I. f1:**
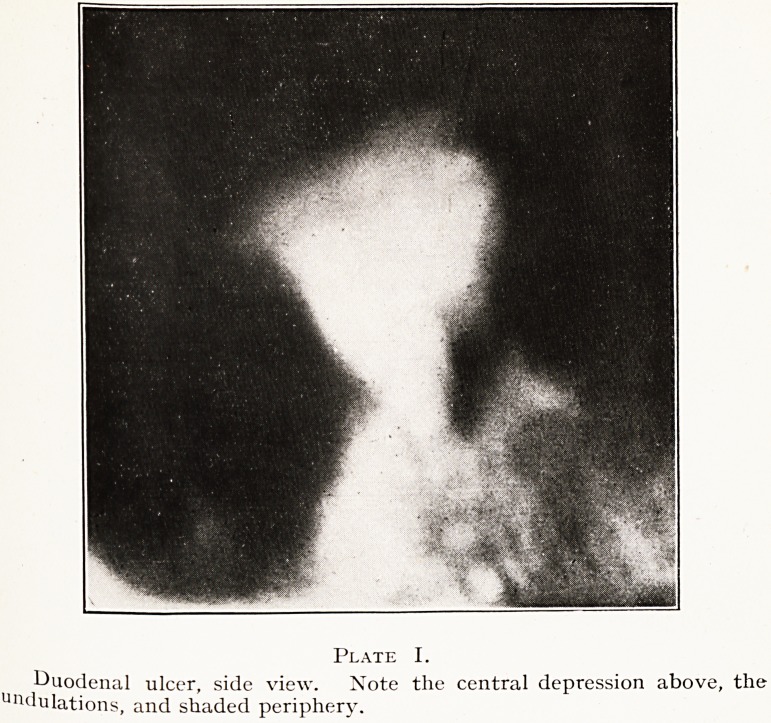


**Plate II. f2:**
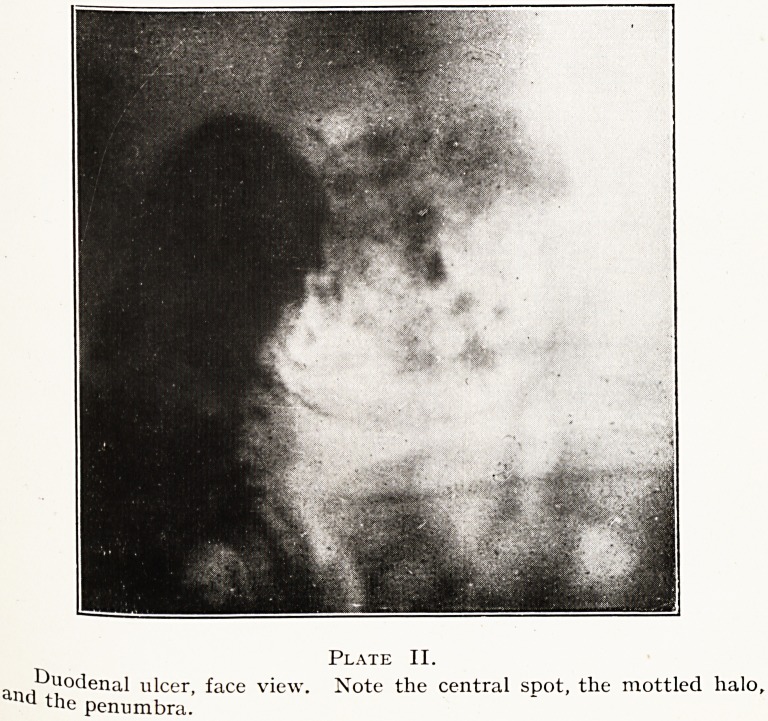


**Plate III. f3:**
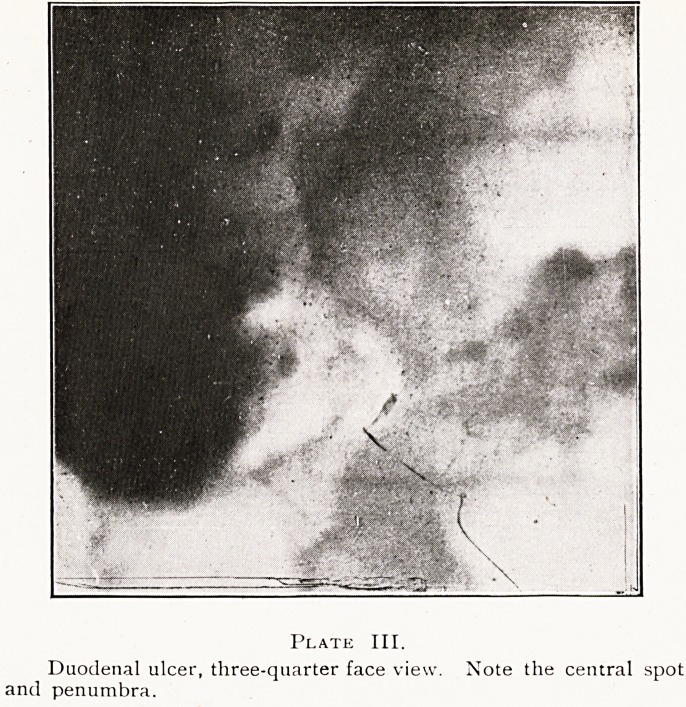


**Plate IV. f4:**
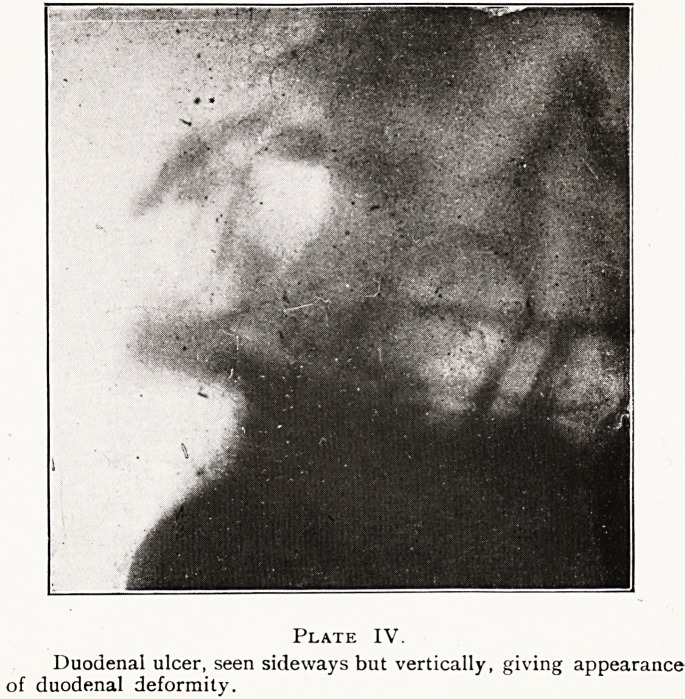


**Fig. 1. Fig. 2. Fig. 3. Fig. 4. f5:**
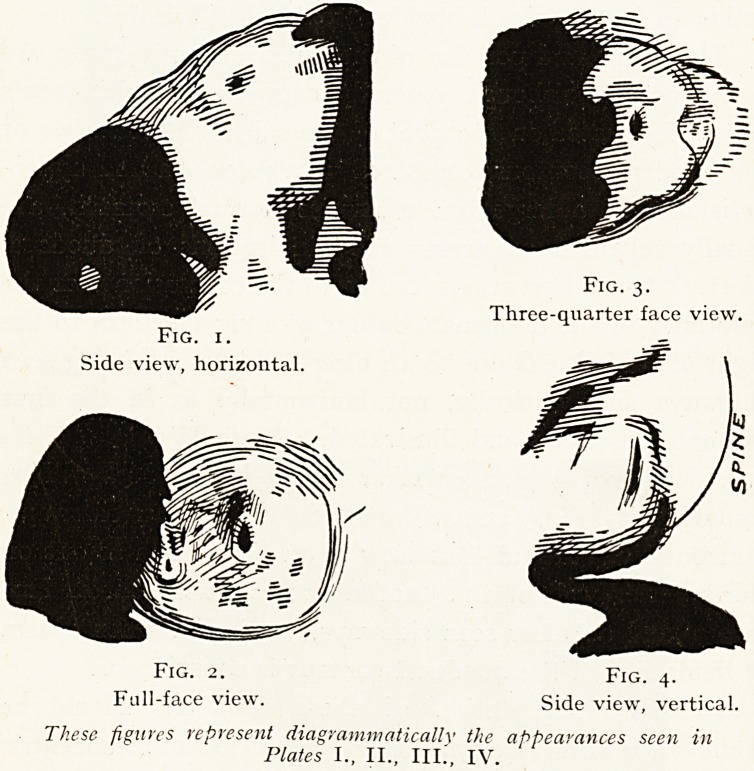


**Plate V. f6:**
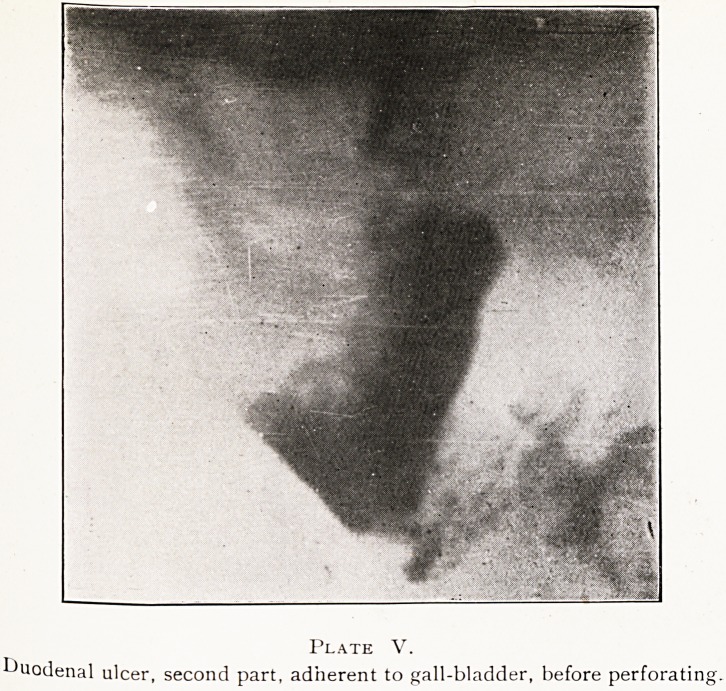


**Plate VI. f7:**